# Subcutaneous furosemide in heart failure: a systematic review

**DOI:** 10.1093/ehjcvp/pvae083

**Published:** 2024-11-08

**Authors:** Joanna Osmanska, Mark C Petrie, Kieran F Docherty, Matthew M Y Lee, John J V McMurray, Ross T Campbell

**Affiliations:** School of Cardiovascular and Metabolic Health, British Heart Foundation Glasgow Cardiovascular Research Centre, University of Glasgow 126 University Place, Glasgow G12 8TA, UK; School of Cardiovascular and Metabolic Health, British Heart Foundation Glasgow Cardiovascular Research Centre, University of Glasgow 126 University Place, Glasgow G12 8TA, UK; School of Cardiovascular and Metabolic Health, British Heart Foundation Glasgow Cardiovascular Research Centre, University of Glasgow 126 University Place, Glasgow G12 8TA, UK; School of Cardiovascular and Metabolic Health, British Heart Foundation Glasgow Cardiovascular Research Centre, University of Glasgow 126 University Place, Glasgow G12 8TA, UK; School of Cardiovascular and Metabolic Health, British Heart Foundation Glasgow Cardiovascular Research Centre, University of Glasgow 126 University Place, Glasgow G12 8TA, UK; School of Cardiovascular and Metabolic Health, British Heart Foundation Glasgow Cardiovascular Research Centre, University of Glasgow 126 University Place, Glasgow G12 8TA, UK

**Keywords:** Heart failure, Diuretics, Furosemide, Subcutaneous, Congestion

## Abstract

**Background and aim:**

Intravenous loop diuretics are the primary treatment for congestion in patients with decompensated heart failure (HF). Furosemide is the most commonly used loop diuretic and is licensed for administration either orally, intramuscularly or intravenously but not subcutaneously. Recently developed, pH-neutral, concentrated, and ‘skin-friendly’ preparations of furosemide have been developed which allow subcutaneous administration. In this systematic review, we summarize and critically appraise the current evidence for subcutaneous furosemide in patients with HF.

**Methods and results:**

The electronic databases MEDLINE, EMBASE, the Cochrane Library, and ClinicalTrials.gov registry were searched up to 30 September 2024. Of the 17 studies identified, 5 were randomized controlled trials (RCTs), 2 were non-randomized controlled studies, 3 were prospective observational cohort studies, and 7 were retrospective observational studies.

All RCTs utilized novel pH-neutral, subcutaneous preparations of furosemide. Bioavailability of novel subcutaneous preparations were similar to intravenous furosemide 10 mg/mL: 99.7% for an 8 mg/mL preparation and 112% for a 30 mg/mL preparation. Natriuresis and diuresis were also similar with novel subcutaneous and conventional intravenous furosemide. Adverse events related to novel preparations included infusion site pain or discomfort, localized skin erythema and minimal swelling. All studies of subcutaneous conventional furosemide were non-randomized with very few data regarding bioavailability or diuretic and natriuretic effect. Subcutaneous conventional furosemide was associated with substantial skin irritation (affecting 3–23% of patients), and skin infections requiring treatment with antibiotics (3–17%).

**Conclusion:**

Novel, pH-neutral preparations of subcutaneous furosemide achieved similar diuresis, natriuresis, and bioavailability to intravenous furosemide, and were well tolerated. Novel preparations may be a treatment option for patients with HF.

## Introduction

‘Congestion’ is a common sequela of heart failure (HF), frequently manifesting as peripheral and pulmonary oedema. Whilst oral diuretics treat and prevent congestion in patients with chronic HF, worsening oedema may necessitate intravenous administration to achieve an adequate clinical response in ‘decompensated’ patients. Intravenous administration commonly requires hospital attendance, with intravenous diuretic being the primary treatment administered in over 90% of patients hospitalized due to HF.^1^

Furosemide has been used for over 5 decades to decongest fluid-overloaded patients and is the most commonly used loop diuretic to treat patients hospitalized with decompensation of HF.^2^ The effects of intravenous administration of furosemide have been extensively studied and reported.^3–8^ More recently, there has been an interest in exploring subcutaneous furosemide administration to facilitate the care of patients with decompensated HF in an outpatient or ambulatory setting.^9^ This approach offers potential advantages for patients (e.g. they can decongest at home) and healthcare systems (e.g. reduced bed occupancy). Conventional furosemide preparations are not licensed for subcutaneous administration, can be associated with skin irritation and patient discomfort, and have limited evidence to support the efficacy of subcutaneous use.^10–13^ Recent advances have been made in the formulations of furosemide, with novel preparations now available. New preparations are concentrated and pH-neutral which overcome issues with the subcutaneous administration of conventional furosemide preparations (due to their alkaline pH).^14–16^ We conducted a systematic review summarizing the current evidence for the use of subcutaneous furosemide in patients with HF.

## Methods

### Search strategy and study selection

This systematic review was conducted according to the Preferred Reporting Items for Systematic Reviews and Meta-Analyses.^17^ The online databases MEDLINE, EMBASE, and the Cochrane Library were searched from their inception to 30th September 2024, using a combination of keywords and Medical Subjects Heading terms. ‘Heart failure’, ‘congestive heart failure’, ‘acute heart failure’, ‘decompensated heart failure’, ‘chronic heart failure’, ‘cardiac failure’, ‘diuretic’, ‘diuretics’, ‘furosemide’, ‘frusemide’, and ‘subcutaneous’ terms were used in the search. Our complete search strategy is presented in Supplementary material online, *Table S1*. Studies and trials of patients with HF receiving subcutaneous furosemide in either inpatient or outpatient settings were included. We included randomized controlled trials (RCTs), non-randomized controlled trials, prospective and retrospective observational cohort studies published in peer reviewed journals and published in English. We excluded studies and trials of non-HF populations, those not describing subcutaneous furosemide, and case series reporting <10 participants. A manual search of references from narrative reviews and trials registered on ClinicalTrials.gov was also performed. Titles and abstracts were screened against the above pre-specified eligibility criteria. Full texts of relevant studies and trials were reviewed by 2 reviewers independently (J.O. and R.T.C.).

### Study quality

The risk of bias and methodological quality of RCTs was assessed using the Cochrane Collaboration risk of bias assessment tool.^18^ For each trial, details of the method of treatment allocation, method of randomization and blinding of investigators and participants and blinding of outcome measurements were assessed. The risk of bias and quality of the individual randomized controlled studies was assessed by 2 investigators independently (J.O. and R.T.C.).

### Data extraction

Methods (study design, inclusion, and exclusion criteria), study population, intervention (preparation of subcutaneous furosemide, dose, other medications), comparator (agent, dose), participants’ clinical characteristics [proportion with HF with reduced ejection fraction (HFrEF) and HF with preserved ejection fraction (HFpEF), left ventricular ejection fraction (LVEF), other HF drugs used], and safety data were collected, where reported. Data pertaining to primary outcomes were collected. We also collected data describing clinical outcomes (mortality, cardiovascular mortality, hospitalizations, HF hospitalizations, and length of stay). We also included surrogate markers of diuretic effect (change in weight, urine output, and natriuresis), as well as bioavailability [including pharmacokinetic (PK) and pharmacodynamic (PD)] data where reported.

### Analysis

The small number of RCTs and the heterogeneity of their design prevented pooled analysis. We elected not to proceed with transformation to a meta-analysis due to the limited number of RCTs with a small number of participants with variable outcomes of interest. The evidence is, therefore, presented as narrative synthesis.

## Results

### Study identification, selection, and quality appraisal

Our search strategy identified 17 studies meeting the selection criteria (Supplementary material online, *Figure S1*): 5 were RCTs, 2 were non-randomized controlled trials with active comparators, 3 were prospective observational cohort studies, and 7 were retrospective observational studies. There was no blinding of treatment allocation in any of the trials (Supplementary material online, *Table S2*).

### Novel (pH neutral) furosemide preparations

Five RCTs which assessed ‘novel’ pH neutral furosemide preparations were identified, all of which had a low or unclear risk of bias (*Table 1* and Supplementary material online, *Tables S2* and *S3*).^14–16,19^

**Table 1:** Randomized controlled trials of novel preparations of subcutaneous furosemide: study design and outcomes

First author/ Year/Country/NCT	*n*/ length of follow up	Setting/ Primary outcome(s)/ Key inclusion criteria	Intervention vs. control	Primary and secondary outcomes results
Konstam^19^ (AT HOME-HF) 2024 US NCT04593823	*n* = 51 30 days	• Outpatient • Win ratio of hierarchical composite of CV mortality/HF events/change in NT-proBNP • Chronic HF, NYHA II/III, signs of congestion	80 mg or 160 mg novel SC furosemide (8 mg/mL) via novel patch infusor pump (Furoscix) over 5 h (mean dose 147±142 mg) vs. usual care with increased dose oral diuretics (mean dose 128±115 mg)	**Primary** **SC vs. oral composite of CV mortality/HF events/change in NT-proBNP:** win ratio 1.11 (95% CI 0.48, 2.5, *P* = 0.806) **Secondary** **SC vs. oral change in weight:** Day 1: −2.0 kg vs. −0.6 kg, *P* = NS Day 2: −3.1 kg vs. −0.9 kg *P* = NS Day 3: −2.9 kg vs. −0.4 kg, *P* = 0.04 Day 17: −3.8 kg vs. −1. 5 kg, *P* = NS Day 30: −3.1 kg vs. −2.1 kg, *P* = NS **SC vs. oral change in 6MWT:** Day 1: 15.8 m vs. −0.8 m, *P* = NS Day 7: 29.3 m vs. 8.3 m, *P* = NS Day 30: 37.6 m vs. −15.1 m, *P* = 0.01 **SC vs. oral change in KCCQ-12:** Day 7: 10 vs. 2.9, *P* = NS Day 30: 16.2 vs. 7.1, *P* = NS
Osmanska^16^ (SQIN-Furosemide PK/PD) 2023 US NCT04384653	*n* = 20^ 24 h	• Outpatient • Bioavailability • Chronic HF, NYHA II/III	80 mg novel SC furosemide (30 mg/mL) via infusion pump over 5 h vs. 80 mg IV conventional furosemide administered as bolus	**Primary** **SC vs. IV bioavailability:** 112% (90% CI: 104, 120%) **Secondary:** **SC vs. IV max plasma concentration:** 2060 ng/mL vs. 13 600 ng/mL **SC vs. IV urine output at 8 h:** 2664 mL vs. 2285 mL **SC vs. IV urine output at 24 h:** 3501 mL vs. 3020 mL **SC vs. IV natriuresis at 8 h:** 7.1 g vs. 6.0 g
Gilotra^15^ 2018 US NCT02579057	*n* = 40 30 days	• Outpatient • Urine output at 6 h • Chronic HF or recent (60 days) HF hospitalization, NYHA II-IV with worsening HF requiring IV diuretics	80 mg novel SC furosemide (8 mg/mL) via infusion pump over 5 h vs. IV conventional furosemide administered as bolus (mean dose 123±47 mg)	**Primary** **SC vs. IV urine output at 6 h:** 1350 mL vs. 1425 mL (*P* = NS) **Secondary:** **SC vs. IV weight loss:** 1.5 kg vs. 1.5 kg (*P* = NS) **SC vs. IV hourly urine output at 6 h:** 325 mL vs. 125 mL (*P* = 0.005) **SC vs. IV natriuresis:** 33 mEq/L vs. 7 mEq/L (*P* = NS) **SC vs. IV 30-day hospitalization rate:** 52% vs. 42% (*P* = NS)
Sica^14^ (FUROPHARM-HF) 2018 Netherlands NCT02350725	*n* = 10 8 h	• Outpatient • Bioavailability (oral to SC) and urine output at 8 h • Chronic HF receiving oral diuretics	80 mg novel SC furosemide (8 mg/mL) via infusion pump over 5 h vs. 80 mg oral furosemide	**Primary** **Oral to SC bioavailability**: 61% **SC vs. oral urine output at 8 h:** 1833 mL vs.1550 mL
Sica^14^ (PK/PD Pivotal study) 2018 US NCT02329834	*n* = 17* 24 h	• Outpatient • Bioavailability (SC to IV), urine output and natriuresis at 8 and 24 h • Chronic HF, NYHA II/III, receiving oral diuretics	80 mg novel SC furosemide (8 mg/mL) via infusion pump over 5 h vs. 80 mg IV conventional furosemide administered as 40 mg bolus followed by another 40 mg bolus 2 h later	**SC vs. IV bioavailability:** 99.7% **SC vs. IV urine output at 8 h:** 2663 mL vs. 2718 mL **SC vs. IV urine output at 24 h:** 3614 mL vs. 3672 mL **SC vs. IV natriuresis at 8 h:** 286 mmol vs. 307 mmol **SC vs. IV natriuresis at 24 h:** 341 mmol vs. 367 mmol

^ Two participants were withdrawn due to inadequate line priming during subcutaneous infusion.

*One participant was withdrawn from the study by investigators before the first administration of the allocated treatment.

6MWT- 6-min walk test; CI, confidence interval; CV, cardiovascular; HF, heart failure; IV, intravenous; KCCQ-12, Kansas City Cardiomyopathy Questionnaire 12; NS, not significant; NT-proBNP, N terminal pro Brain type natriuretic peptide; NYHA, New York Heart Association; PD, pharmacodynamics; PK, pharmacokinetics; SC, subcutaneous.

The studies describing novel preparations of furosemide were all published between 2018 and 2024. Two novel preparations of furosemide were described, both of which utilize a buffer and active medicinal product (furosemide) combination, which together are pH neutral. The dose used in each study, for both preparations was 80 mg, and both were designed to be administered via a subcutaneous infusion over 5 h. Both preparations were delivered using a biphasic infusion with 30 mg delivered over the first hour and 12.5 mg/h for the following 4 h. The preparations described had different concentrations of furosemide; the SQ Innovation Inc. preparation was more concentrated than conventional furosemide at 30 mg/mL (80 mg in 2.7 mL) and the scPharmaceuticals^®^ preparation was similar to conventional furosemide at 8 mg/mL (80 mg in 10 mL). The conventional preparation of ‘neat’ furosemide used as a comparator in the RCTs had a concentration of 10 mg/mL (80 mg in 8 mL).

All of the RCTs were early phase and small (10–51 participants), enrolled a total of 138 patients in an outpatient setting, and included an arm with a novel preparation of furosemide designed for subcutaneous administration. One of these RCTs included participants with worsening HF who required treatment with intravenous diuretics, and were treated in an ambulatory facility,^15^ and a second included outpatients with worsening HF requiring intensification of diuresis.^19^

**Figure 1:**
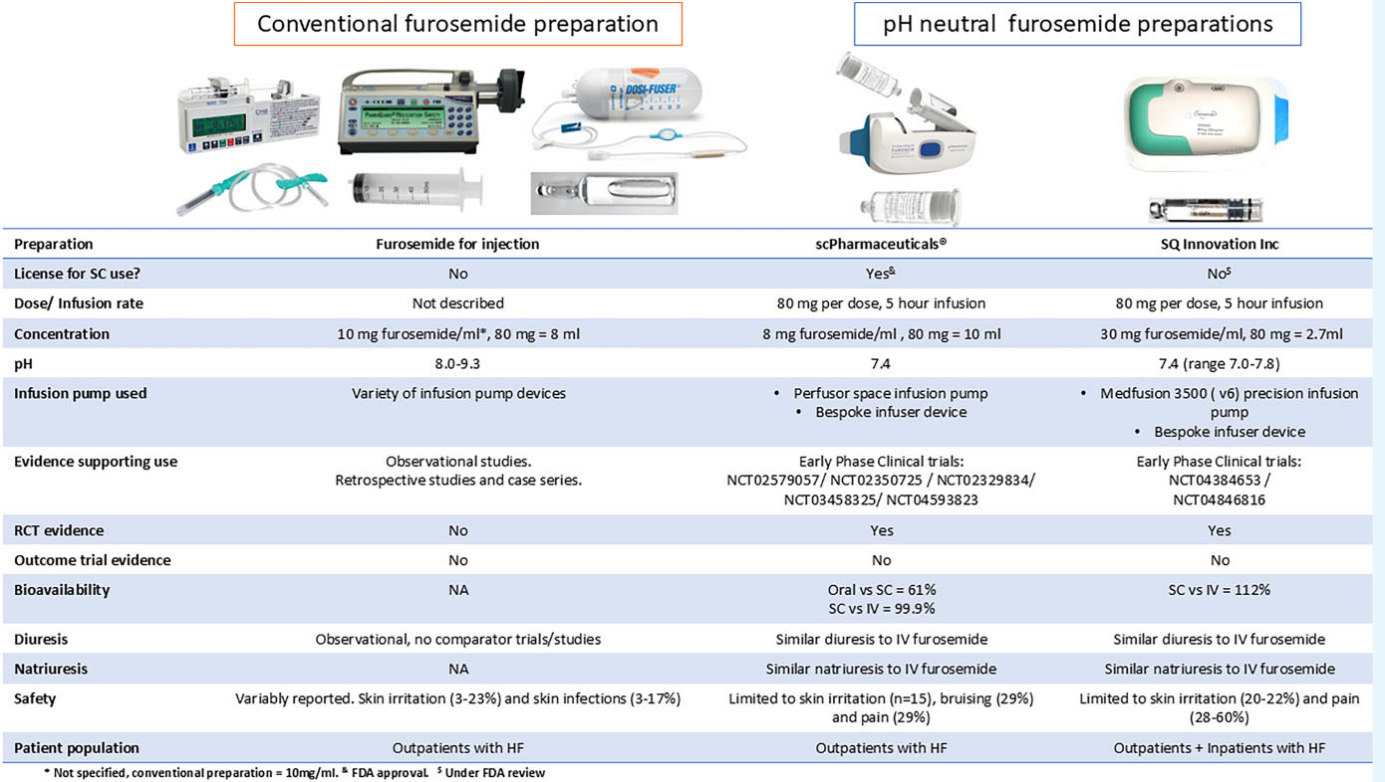
Comparison of subcutaneous furosemide preparations.

Two non-randomized, observational studies assessed novel furosemide preparations.^16,20^ One of these studies described the use, safety, tolerability, and on-body performance of a bespoke patch-infusor device designed to deliver pH neutral furosemide (SQ Innovation Inc, 30 mg/mL, and 80 mg in 2.7 mL) in 20 patients being treated for decompensated HF in hospital (*Figure 1*).^16^ The second observational study, also recruited inpatients being treated for decompensated HF, and described the use of another bespoke patch-infusor device designed to deliver pH neutral furosemide (scPharmaceuticals^®^, 8 mg/mL, and 80 mg in 10 mL).^20^

Four of the RCTs utilized Food and Drug Administration (FDA) approved and CE marked, commercially available infusion pumps to deliver both novel and conventional preparations of furosemide. One RCT utilized an FDA approved bespoke patch infuser device.^19^

#### Patients

Median age ranged between 56 and 75 years (range of mean age 57–71). The majority of patients included were male, with the exception of one RCT which enrolled patients with worsening HF.^15^ Patients were eligible for the trials and studies of novel furosemide regardless of LVEF. LVEF was only reported in 2 of the 5 RCTs and 1 of 2 observational studies identified, with most patients having HFrEF.^15,16^ All of the trials and studies of novel furosemide preparations excluded patients with advanced renal disease (Supplementary material online, *Table S5*), although one trial included patients with an estimated glomerular filtration rate (eGFR) ≥20 mL/min/1.73m^2^ (median eGFR in subcutaneous group 55.0 ± 21.1 mL/min/1.73m^2^).^19^ The baseline eGFR ranged between a mean of 45–62 mL/min/1.73m^2^ (range of median 54–63 mL/min/1.73m^2^). New York Heart Association (NYHA) class was recorded in all but one of the identified trials/studies.^20^ Median N-terminal pro-B-type natriuretic peptide (NT-proBNP) ranged from 897 to 5184 pg/mL. Use of disease modifying HF pharmacotherapy was higher in the trials of outpatient/stable than those of inpatients/decompensated HF. Two trials did not report baseline HF disease modifying therapy use at baseline.^14^

#### Bioavailability

Three of the trials assessed the bioavailability of novel pH neutral furosemide preparations compared to conventional furosemide (intravenous and oral preparations), see *Table 1*.^14,16^ The SQIN-Furosemide PK/PD trial (NCT04384653) randomized patients to receive a single dose of 80 mg of intravenous furosemide (10 mg/mL) or 80 mg of subcutaneous furosemide (30 mg/mL, SQ Innovation Inc) administered with a conventional subcutaneous infusion pump. Treatment with the subcutaneous preparation of furosemide resulted in similar bioavailability [112%, 90% confidence interval (CI): 104, 120%] as the same dose administered intravenously as a bolus.^16^

The Furosemide Pharmacodynamics and Pharmacokinetics After Subcutaneous or Oral Administration (FUROPHARM-HF; NCT02350725) trial randomized 10 patients to treatment with either the novel subcutaneous furosemide (8 mg/mL, scPharmaceuticals^®^) preparation or oral furosemide with follow-up of 8 h. Oral administration of furosemide resulted in lower bioavailability, with a mean of 61% (range 33–96%) when compared to subcutaneous furosemide. The Crossover Study to Compare the Pharmacokinetics and Bioavailability of a Novel Furosemide Regimen Administered Subcutaneously vs. the Same Dose Administered Intravenously in Subjects with Chronic HF (PK/PD Pivotal study; NCT02329834) study randomized 17 patients to the novel subcutaneous furosemide (8 mg/mL, scPharmaceuticals^®^) preparation or intravenous furosemide.^14^ The mean bioavailability was 99.7% in comparison to intravenous furosemide administered as two 40 mg boluses administered 2 h apart. Although bioavailability was not formally assessed in the SUBCUT-HF I (A Study to Assess Safety and Efficacy of a Novel Patch Infusor Device and Novel SUBCUTaneous Furosemide Formulation Combination in Patients With Heart Failure) study (NCT 04846816), plasma furosemide levels were measured and all participants achieved a therapeutic plasma concentration at 60 min (the median plasma furosemide concentration 1155 ng/mL, IQR 848–1665 ng/mL).^16^

#### Diuretic/natriuretic effect

The diuretic effect of a novel pH neutral furosemide preparation was assessed as the primary endpoint in 1 trial (total urine volume at 6 h), and recorded as secondary endpoint(s) in the other 3 RCTs and 1 observational study (total urinary output and natriuresis at 8 and 24 h), see *Table 1*.^14–16^ Weight loss was assessed as a surrogate of diuresis in one trial.^19^ Diuresis and natriuresis were either similar or higher following treatment with the novel preparation of subcutaneous furosemide preparation when compared with conventional furosemide preparations (*Table 1*). In the Sub-Q Versus IV Furosemide in Acute Heart Failure trial (NCT02579057) there was no difference in the median urine output between the 2 treatment arms. The total concentration of urinary sodium was higher with the subcutaneous (scPharmaceuticals^®^, 8 mg/mL) than intravenous furosemide, (although the report suggests wide standard deviations and that the results did not reach statistical significance). There was no difference in weight loss between the two groups. Similar equivalence in diuretic effect (as assessed by total urinary output) was shown in the two PK/PD trials of this pH neutral formulation of furosemide (scPharmaceuticals^®^, 8 mg/mL).^14^ Cumulative urine output over 8 h was similar between the subcutaneous and oral furosemide. Similarly, there was no difference between subcutaneous and intravenous furosemide at 8 or 24 h. There was no difference in natriuresis (assessed by total urinary sodium excretion) between subcutaneous and intravenous furosemide at 8 h or 24 h. The AT HOME-HF trial (Avoiding Treatment in the Hospital With Furoscix for the Management of Congestion in Heart Failure—A Pilot Study, NCT04593823) randomized outpatients with worsening HF 2:1 to either treatment with subcutaneous furosemide (80 mg once or twice daily, scPharmaceuticals®, 8 mg/mL) or usual care (escalation of oral diuretic regime), although there was numerically greater weight change this only reached statistical significance at day 3 of 30.^19^ The SQIN-Furosemide PK/PD trial demonstrated similar diuresis but greater natriuresis with subcutaneous (30 mg/mL, SQ Innovation Inc) vs. intravenous furosemide (10 mg/mL). Urine output was similar following treatment with subcutaneous furosemide compared to intravenous furosemide at 8 and 24 h. Treatment with subcutaneous furosemide resulted in greater natriuresis than intravenous furosemide at 8 h.

#### Safety and tolerability

The safety of subcutaneous furosemide was assessed in all the RCTs (Supplementary material online, *Table S6*). Overall, the novel pH neutral furosemide preparations were well tolerated. One of the RCTs reported no adverse events in 40 participants, following treatment with either subcutaneous (scPharmaceuticals^®^, 8 mg/mL) or intravenous furosemide.^15^ In the SQIN-Furosemide PK/PD study of 20 patients, there were 4 adverse events, of which 2 were attributed to subcutaneous furosemide (SQ Innovation Inc, 30 mg/ml): pain at the infusion site and a transient episode of orthostatic hypotension. Five (28%) participants reported pain or discomfort during treatment with subcutaneous furosemide, and local skin reactions were noted in 4 (22%) participants treated with subcutaneous furosemide.^16^ The FUROPHARM-HF and PK/PD Pivotal studies reported 15 adverse events in 27 patients who received subcutaneous furosemide (scPharmaceuticals^®^, 8 mg/mL) in both studies.^14^ The reported adverse events were limited to 9 cases of ‘very slight erythema’ and 6 episodes of minimal swelling at the site of injection during or after completion of treatment with novel subcutaneous furosemide. Additionally, 1 patient experienced ‘well-defined erythema’ during IV infusion of conventional furosemide.^14^ Adverse events were common in the AT HOME HF pilot RCT, with 23 (67%) of the subcutaneous and 9 (53%) of the usual care arms having at least one AE. These included hypokalaemia (21% vs. 0%), renal impairment (15% vs. 18%), infusion site pain (15% vs. 0%), and fatigue (3% vs. 12%), in subcutaneous vs. usual care, respectively. Serious AEs were reported in 35% of subcutaneous and 24% of usual care groups, none of which were deemed related to treatment with furosemide. There was no statistical difference in HF hospitalizations. There was one sudden cardiac death at day 30 in the subcutaneous arm, deemed not related to study treatment.

In the non-randomized studies of novel subcutaneous preparations of furosemide, adverse events were common. There were no serious adverse events. In the SUBCUT-HF I trial of 20 patients, a local skin reaction (transient erythema) occurred in 4 (20%) participants and 12 (60%) reported pain during treatment, with 8 (40%) reporting discomfort limited to needle insertion only.^16^ In FREEDOM-HF, in 24 patients who were treated with the less concentrated novel preparation of subcutaneous furosemide (scPharmaceuticals^®^, 8 mg/mL) localized skin reactions were common but limited to infusion site bruising (29%) and pain (29%).^20^

### Conventional furosemide preparations

Our search identified 10 non-randomized studies describing the use of conventional furosemide administered subcutaneously: 3 were prospective observational cohort studies and 7 were retrospective observational cohort studies. These were heterogeneous in design and small, the largest of which included 116 patients (*Table 2* and Supplementary material online, *Table S4*). There were no RCTs, and only 3 studies included a comparator arm. There were no PK/PD data describing the use of conventional furosemide administered subcutaneously reported in any of the studies. 6 of the studies reported clinical outcomes such as mortality and HF hospitalization, however, there were no power calculations provided in relation to sample size for these endpoints and the studies. Three studies described the diuretic effect of the administration of conventional furosemide subcutaneously using change in weight as a surrogate marker.^23–25^ These studies reported weight loss associate with the use of subcutaneous furosemide over a variable time period. The description of participants was variable (Supplementary material online, *Table S4*), with key HF characteristics such as concomitant therapy, NT-proBNP, renal function, frequently not reported.

**Table 2:** Non-randomized studies of novel and conventional preparations of furosemide administered subcutaneously: study design and outcomes

First author/ Trial/ Year/Country	*n*/ length of follow up	Setting/ Primary outcome(s)/ Key inclusion criteria	Preparation of SC furosemide/ mean daily dose [mg]	Comparator/ mean daily dose [mg]	Primary outcome result
**Non-randomized controlled**					
Lopez-Vilella^21^ 2021 Spain	• *n* = 27 (10 SC and 17 oral)* • 3 years	• Outpatient • Unscheduled attendance at 30 days • Diuretic resistance, NYHA III-IV	Conventional furosemide, delivered via an elastomeric pump, 5 days treatment 100 mg	Oral furosemide solution, 250 mg for 5 days	**Unscheduled attendance at 30 days:** SC 10% vs. oral 24% (*P* = NS)
Austin^34^ 2013 UK	• *n* = 25 (11 SC and 14 IV)* • Up to 59 days	• Outpatient • Change between pre- and post-treatment ESAS mean score, Change between pre- and post-treatment MNA mean score, Service satisfaction (VAS) • NYHA III/IV, Evidence of fluid overload (>3 kg from baseline)	Conventional furosemide delivered via a syringe driver, dose NR	IV furosemide, dose NR	**Change between pre- and post-treatment ESAS mean score:** −11.7 (*P* = 0.001)^ **Change between pre- and post-treatment MNA mean score**: NS^ **Service satisfaction (VAS):** *n* = 10 90–100% satisfaction^
**Prospective observational cohort**					
Osmanska^16^ (SUBCUT-HF I) 2023 UK NCT04846816	• *n* = 20 • 24 h	• Inpatient • Treatment related adverse events, Infusion site pain, Device performance, Pharmacokinetic parameters • Decompensated HF, Requiring treatment with IV furosemide 40–200 mg	80 mg novel SC furosemide (30 mg/mL) via novel patch infusor pump	N/A	**Treatment related adverse events:** None **Infusion site pain:** 60% reported pain at the time of injection **Device performance:** 1 dressing became loose and the device detached from the skin **Pharmacokinetic parameters:** Median plasma furosemide concentration at 60 min: 1155 ng/mL
FREEDOM-HF^20^ 2023 US NCT03458325	• *n* = 24 • 30 days	• Outpatient • Difference in heart failure- related healthcare utilization costs • Decompensated HF, NYHA II-III, Treated with 40–160 mg oral furosemide (or equivalent)	80 mg novel furosemide (8 mg/mL) via novel patch infusor pump (Furoscix)	N/A	**Difference in heart failure- related healthcare utilization costs:** Mean difference SC vs. IV: –$16995.3 (95% CI: –22 187.9; -11802.70, *P* < 0.001)
Lozano Bahamonde^25^ 2018 Spain	• *n* = 12 • Mean follow up: 203 days	• Outpatient • The rate of HF hospitalization • Decompensated HF not responding to oral diuretics or repetitive use of IV furosemide	Conventional furosemide, delivered via a single-use elastomeric pump 135 mg	N/A	**The rate of HF hospitalization:** 17%
**Retrospective observational cohort**					
Birch^22^ 2023 UK	• *n* = 116 (130 episodes) • Median follow up: 10 days	• Outpatient • Hospice/community hospital • HF patients known to palliative-cardiology service	Conventional furosemide delivered via a syringe driver Starting dose 125 mg, End of treatment dose 137 mg	N/A	**Weight change:** Median −4 kg **Patient reported breathlessness score:** lower by 3
Brown^13^ 2022	• *n* = 28 (36 consecutive episodes) • Follow up not reported	• Outpatient • Rate of hospitalization in comparison to 6 months prior to treatment, Anticipated to be in final 12 months of life, on GP palliative care register, on maximal oral diuretic dose	Conventional furosemide, delivered via a syringe driver Median 200 mg	N/A	**Rate of hospitalization in comparison to 6 months prior to treatment:** lower from 2.87 to 0.73 (95% CI:1.35–2.91, *P* < 0.001)
Civera^26^ 2022 Spain	• *n* = 55 • 30 days	• Outpatient • Change in NYHA class at 72 h and 30 days, Changes in dyspnoea VAS at 72 h and 30 days, Change in lnNT-proBNP at 72 h and 30 days, Change in pedal oedema grading scale at 72 h and 30 days, Change in weight at 72 h and 30 days, Change in lnCA-125 at 72 h and 30 days, Change in urinary sodium at 24 h, 48 h, 72 h and 30 days*^&^* • Structural or functional cardiac abnormality and/or NT-proBNP >1000 pg/mL and/or • Previous treatment with loop diuretics for HF	Conventional furosemide, delivered via a single-use continuous infusion pump system (DOSI-FUSER) Median 100 mg	N/A	**Change in NYHA class at 72 h and 30 days** ***^&^*** 72 h: −0.6, *P* < 0.001 30 days: −0.7, *P* < 0.001 **Changes in dyspnoea VAS at 72 h and 30 days*****^&^*** 72 h: −2.2 *P* < 0.001 30 days: −3.8, *P* < 0.001 **Change in lnNT-proBNP at 72 h and 30 days*****^&^*** 72 h: −0.24, *P* = 0.02 30 days: −0.35, *P* < 0.001 **Change in pedal oedema grading scale at 72 h and 30 days*****^&^*** 72 h: −1.1, *P* < 0.001 30 days: −1.5, *P* < 0.001 **Change in weight at 72 h and 30 days*****^&^*** 72 h: −3.2 kg, *P* < 0.001 30 days: −3.6 kg, *P* < 0.001 **Change in lnCA-125 at 72 h and 30 days*****^&^*** 72 h: 0.06, *P* = NS 30 days: −0.27, *P* < 0.001 **Change in urinary sodium at 24 h, 48 h, 72 h and 30 days*****^&^*** 24 h: 31 mmol/L, *P* < 0.001 48h: 30 mmol/L, *P* < 0.001 72 h: 22 mmol/L, *P* < 0.001 30 days: −1 mmol/L, *P* = NS
Lozano Bahamonde^24^ 2019 Spain	• *n* = 16 (12 with congestive symptoms, 4 euvolaemic*) • Mean follow up: 160 days	• Outpatient • Rate of HF hospitalization (in comparison to 12 months prior to treatment) • Decompensated HF, Refractory to oral furosemide, Requiring treatment with IV furosemide or ≥2 HF hospitalizations in 6 months (euvolemic group*)	Conventional furosemide, delivered via an elastomeric pump Congested patients: 234 mg Euvolemic patients: 123 mg	N/A	**Rate of HF hospitalization (in comparison to 12 months prior to treatment)** 12 months before treatment: 0.26 hospitalization/patient/month After treatment: 0.02 hospitalization/patient/month
Galindo-Ocana^27^ 2013 Spain	• *n* = 44 (17 SC and 27 IV, 97 episodes) • 1 year	• Outpatient (SC)/Hospital (IV) • Median length of stay in hospital per episode, Mortality, Median survival time • Patients with decompensated HF consecutively attended to at home, NYHA III-IV	Conventional furosemide (method of delivery not reported) 160 mg	IV furosemide median dose 104 mg (IQR 80-171 mg)	**Median length of stay in hospital per episode:** SC 13 days vs. IV 7 days (*P* = 0.001) **Mortality:** SC 79% vs. IV 96% (*P* = NS) **Median survival time:** SC 27 days vs. IV 18 days (*P* = NS)
Zatarain-Nicolas^23^ 2013 Spain	• *n* = 24 (41 episodes) • Mean follow up: 9 days	• Outpatient • Weight change • Patients with decompensated HF requiring parenteral diuretics	Conventional furosemide, delivered via elastomeric pump 146 mg	N/A	**Weight change:** Lower from 79 to 77 kg (*P* < 0.001)
Zacharias^10^ 2011 UK	• *n* = 32 (43 consecutive episodes) • Median follow up: 11 days	• Hospital/outpatient/hospice • The rate of prevention of hospital admission/transfer for parenteral diuretics, Weight loss, The rate of prevention of symptoms during the dying phase • Advanced HF patients referred to palliative-cardiology service	Conventional furosemide delivered via syringe driver Outpatients 142 mg, Hospital 109 mg, Hospice 138 mg	N/A	**The rate of prevention of hospital admission/transfer for parenteral diuretics:** 93% **Weight loss:** Median 5.6 kg **The rate of prevention of symptoms during the dying phase:** 100%

^Results of IV treatment arm only, *n* = 11.

*Euvolaemic group included patients with heart failure with at least 2 hospital admissions in preceding 6 month.

& Multivariate adjusted for age, sex, baseline estimated glomerular filtration rate, left ventricular ejection fraction, and baseline endpoint value.

A&E, accident and emergency; BMI, body mass index; eGFR, estimated glomerular filtration rate; ESAS, Edmonton Symptom Assessment System; GP, general practitioner; HF, heart failure; IV, intravenous; lnCA-125, natural logarithm of cancer antigen 125; lnNT-proBNP, natural logarithm of N-terminal pro-B-type natriuretic peptide; MNA, Mini Nutritional Assessment; N/A, not applicable; NR, not reported; NS, not significant; NT-proBNP, N-terminal pro-B-type natriuretic peptide; NYHA, New York Heart Association; OMT, optimal medical therapy; PD, pharmacodynamics; PK, pharmacokinetics; SBP, systolic blood pressure; SC, subcutaneous; VAS, visual analogue scale.

There was only one study identified where patients were prospectively enrolled and provided informed consent. This study approached consecutive outpatients at a single centre in Spain with advanced HF and diuretic resistance, who were not candidates for alternative therapy. Patients were treated with either a subcutaneous infusion of conventional furosemide (*n* = 10; prepared at a concentration of 2.1 mg/mL using 0.9% sodium chloride solution), administered at a dose of 100 mg over 24 h, or oral furosemide solution (*n* = 17; prepared from the same 500 mg per 50 mL ampoules as the subcutaneous formulation with a half ampoule administered every 12 h). The decision to provide either treatment was based upon a patient's home location to the treatment centre. Both treatments were given for 5 days. There was no difference in the rates of unscheduled HF attendance at 30 days between the two treatments, although the study was not adequately powered to.^21^

#### Safety and tolerability

Reporting of adverse events was variable in studies describing the use of conventional furosemide administered subcutaneously (Supplementary material online, *Table S6*). Local infusion site adverse reactions were frequently reported, with skin reactions or irritation observed in 3–23% of patients.^10,21–23^ Skin reactions requiring treatment with antibiotics were also commonly reported with 3–17% patients affected.^10,13,24–27^ The other individually reported adverse events included 3 hematomas,^27^ 2 skin erosions,^24^ 1 abscess formation requiring surgical drainage,^23^ 1 case of hypokalaemia (K < 3.0 mmol/L),^23^ and 1 case of treatment withdrawal due to unspecified local complication.^25^

## Discussion

There were two main findings: (i) novel preparations of subcutaneous furosemide resulted in a similar diuresis and natriuresis when compared to conventional furosemide administered orally or intravenously and are well tolerated with low numbers of adverse effects. (ii) there were limited data reporting the diuretic and natriuretic effects of conventional subcutaneous furosemide and adverse events in terms of skin irritation, localized skin reactions and infections were frequent.

Conventional furosemide has been licensed for use in oral and intravenous and intramuscular (but not subcutaneous) forms for over 50 years. Furosemide is highly acidic (pH 3.9) and insoluble at physiological pH. The conventional preparations of furosemide which are licensed for parenteral administration are prepared using NaOH, resulting in a pH of 8.0–9.3.^28^ This results in skin irritation and discomfort at the injection site.

Despite the lack of licensing, conventional furosemide is administered ‘off-licence’ with reports of subcutaneous administration of furosemide in patients with HF dating from 20 years ago.^12^ In the studies identified in our review, subcutaneous use of conventional furosemide (large volumes administered by conventional pumps) was associated with frequent adverse events and localized skin reactions, affecting nearly 1 in every 4 patients. Skin infections requiring treatment with antibiotics were common, and a more serious infection requiring surgical intervention for abscess drainage was also reported. These undesirable side effects limit the potential benefit and usability of subcutaneous administration of conventional furosemide preparations in current clinical practice.

Two novel formulations of furosemide with a physiologic pH of 7.4, which can be administered subcutaneously (scPharmaceuticals^®^, 8 mg/mL and SQ Innovation Inc, 30 mg/mL), have been developed.^14–16^ These formulations have similar bioavailability and similar diuretic and natriuretic effects to conventional furosemide administered intravenously, with a favourable safety profile. Both preparations are designed to be administered over 5 h with custom-made devices. These patch pumps have no extra tubing, which means the pump is attached directly to the skin with an adhesive layer, making it easier to use and more discrete.^16,29–31^

There are no RCTs comparing conventional furosemide administered subcutaneously versus intravenously. Justification of the subcutaneous administration of conventional furosemide comes from a limited number of non-randomized studies, which have recently been systematically reviewed.^32,33^ The participants included in these studies had variable clinical characteristics, with a broad spectrum of symptoms and severity of HF, ranging from those with chronic HF to those in the palliative phase of the disease, and were small and heterogenous in design.

Our systematic review demonstrates that robust evidence to support the subcutaneous administration of conventional furosemide is limited. Recent advances in the development of novel, skin-friendly, subcutaneous formulations of furosemide are encouraging, with 4 small RCTs reporting similar bioavailability of novel preparations of subcutaneous furosemide when compared to oral and intravenous conventional furosemide, with few adverse side effects.^14–16^

There is a requirement for more evidence to investigate the role of subcutaneous furosemide in routine community or ambulatory heart failure clinical care. Specifically, questions remain about how subcutaneous furosemide could be utilized as part of a wider strategy for managing decompensated HF: Which patients can be managed with subcutaneous furosemide in the community safely and effectively? Can patients hospitalized with congestion safely be discharged to diurese at home? Is this cost effective? Can hospitalizations be prevented? Are there different treatment effects between different phenotypes, such as HFrEF vs. HFpEF, patients with renal disease or patients requiring different doses of loop diuretics? Larger RCTs are required to answer these questions. The ongoing multicentre SUBCUT-HF II trial (the Use of a Novel SUBCUTaneous Preparation of Furosemide to Facilitate Early Supported Discharge of Patients With Heart Failure, NCT05419115) will investigate whether a combination of pH-neutral, concentrated furosemide, administered via a patch infusor device, can facilitate an ambulatory care pathway for patients with HF.

### Limitations

There was considerable heterogeneity across the studies and trials in patient characteristics, symptom severity, diuretic doses, and formulations of furosemide used. The number of patients was small (the largest RCT included 51 patients and the largest observational study included 116 patients) and studies reported variable outcomes. High doses of furosemide were not tested in the RCTs, and there were relatively few patients with low eGFR. Data on patient characteristics, comorbidities, and treatments for HF were infrequently reported. Only one of the RCTs described patient-reported outcome measures, although the number of participants was too small to make meaningful comparisons.^19^ Future trials should include these, particularly comparing comfort and ease of use between subcutaneous and intravenous preparations where possible.

## Summary

Subcutaneous administration of conventional furosemide is unlicensed, limited by its pharmacological properties and is associated with frequent adverse effects. Studies and early phase trials of subcutaneous administration of novel, pH-neutral, skin-friendly preparations have reported similar bioavailability, diuresis and natriuresis to intravenous furosemide as well as good tolerability. Further evidence is required to investigate the use the role of novel formulations in routine clinical practice.

## Supplementary Material

pvae083_Supplemental_Files

## Data Availability

The data underlying this article will be shared on reasonable request to the corresponding author.
