# Supplementary material for: Subcutaneous furosemide in heart failure: a systematic review
Source: Eur Heart J Cardiovasc Pharmacother. 2024 Nov 8;11(1):94–104. doi: 10.1093/ehjcvp/pvae083 (PMC11805693; doi:10.1093/ehjcvp/pvae083)
Supplement: pvae083_Supplemental_Files [file pvae083_supplemental_files.zip › Supplementary Table 1 clean.docx]

## Table S1 A: Literature search Embase + Medline search keywords.

|  | Search terms | n |
| --- | --- | --- |
| 1 | (heart failure or congestive heart failure or acute heart failure or decompensated heart failure or chronic heart failure or cardiac failure).mp. [mp=ti, ab, hw, tn, ot, dm, mf, dv, kf, fx, dq, bt, nm, ox, px, rx, an, ui, sy, ux, mx] | 857604 |
| 2 | (diuretic or diuretics or furosemide or frusemide).mp. [mp=ti, ab, hw, tn, ot, dm, mf, dv, kf, fx, dq, bt, nm, ox, px, rx, an, ui, sy, ux, mx] | 286075 |
| 3 | subcutaneous.mp. [mp=ti, ab, hw, tn, ot, dm, mf, dv, kf, fx, dq, bt, nm, ox, px, rx, an, ui, sy, ux, mx] | 685133 |
| 4 | 2 and 3 | 5236 |
| 5 | 1 and 4 | 1231 |
| 6 | limit 5 to English language | 1183 |
| 7 | limit 6 to human | 1060 |
| 8 | limit 7 to humans | 1060 |
|  | ab= abstract; an= accession number; bt= book title; dm= device manufacturer; dq= candidate term word; dv= device trade name; fx= floating sub-heading word; hw= heading word; kf= keyword heading word; kw= keywords; mf= drug manufacturer; mp= multi-purpose; mx= anatomy supplementary concept word; nm= name of substance word; ot= original title; ox= organism supplementary concept word; px= protocol supplementary concept word; rx= rare disease supplementary concept word; sh= subject heading; sy= synonym ; ti= title; tn= trade name; ui= unique identifier; ux= population supplementary concept word |  |

## Table S1 B: Literature search Cochrane keywords.

|  | Search terms | n |
| --- | --- | --- |
| 1 | (heart failure):ti,ab,kw OR (congestive heart failure):ti,ab,kw OR (acute heart failure):ti,ab,kw OR (decompensated heart failure):ti,ab,kw OR (chronic heart failure):ti,ab,kw (Word variations have been searched) | 46052 |
| 2 | (diuretic or diuretics or furosemide or frusemide). ti,ab,kw | 3063 |
| 3 | (subcutaneous):ti,ab,kw | 39179 |
| 4 | 1 and 2 | 1044 |
| 5 | 4 and 3 | 27 |
|  | ab= abstract; kf= keyword heading word; kw= keywords; mf= drug manufacturer; mp= multi-purpose; ti= title; |  |

Excel spreadsheets were employed as data extraction forms and populated directly from the main publication. For each study, summary data were extracted from the main publication and appendices by one author (JO and verified independently by two authors (RTC and MML).
