# Supplementary material for: Subcutaneous furosemide in heart failure: a systematic review
Source: Eur Heart J Cardiovasc Pharmacother. 2024 Nov 8;11(1):94–104. doi: 10.1093/ehjcvp/pvae083 (PMC11805693; doi:10.1093/ehjcvp/pvae083)
Supplement: pvae083_Supplemental_Files [file pvae083_supplemental_files.zip › Supplementary Table 2 clean.docx]

## Table S2: Bias assessment of randomised controlled trials included in the review.

| **Konstam (AT HOME-HF) 2024** | | |
| --- | --- | --- |
| Methods | 30 days, randomised 2:1 ratio | |
| Participants | 51, all completed study, SC treatment: mean (SD) age 74 (11) years, NYHA II-III | |
| Interventions | Novel preparation of SC furosemide (30 mg/ml) vs. usual care with increased dose oral diuretics | |
| Outcomes | Win ratio of hierarchical composite of CV mortality/HF events/change in NT-proBNP. | |
| **Risk of bias** |  | |
| **Bias** | **Authors’ judgement** | **Support for judgement** |
| Random sequence generation (selection bias) | Low risk | Participants were randomly allocated in 1 2:1 ratio to each treatment arm  Participants recruitment and randomisation processes were not described |
| Allocation concealment (selection bias) | Unclear risk | No blinding to treatment allocation  The randomisation process was not described  The blinding of outcome assessors not described  The sequence was predictable for the last participant |
| Blinding of participants and personnel (performance bias) | Unclear risk | Participants and investigators were not blinded  Different schedule of assessments for each treatments arm (including safety assessments)  Some of the outcomes were self-reported by the participants |
| Blinding of outcome assessment (detection bias) | Unclear risk | Participants were not blinded  The blinding of outcome assessors was not described  Some of the outcomes were self-reported by the participants  Laboratory testing was performed at the central laboratory |
| Incomplete outcome data (attrition bias) | Low risk | Change to diuretic treatments not reported |
| Selective reporting (reporting bias) | Low risk | The full study protocol is unavailable |
| Other bias | Low risk | 41% of patients in usual care continued with the same dose of oral diuretics |
| **Osmanska**^16^ **(SQIN-Furosemide PK/PD) 2023** | | |
| Methods | 24 hours, randomised, crossover | |
| Participants | 20, 2 excluded, median (IQR) age 71 (64-74) years), NYHA II-III | |
| Interventions | Novel preparation of SC furosemide (30 mg/ml) vs. IV conventional furosemide | |
| Outcomes | PK/PD parameters: SC furosemide bioavailability was 112% | |
| **Risk of bias** |  | |
| **Bias** | **Authors’ judgement** | **Support for judgement** |
| Random sequence generation (selection bias) | Low risk | Participants were randomly allocated in a 1:1 ratio to each treatment arm  Two participants were withdrawn from the study due to problems with line priming  Participants recruitment and randomisation processes were not described |
| Allocation concealment (selection bias) | Unclear risk | The randomisation process was not described  The blinding of outcome assessors not described  The sequence was predictable for the last participant |
| Blinding of participants and personnel (performance bias) | Unclear risk | Participants were not blinded  The blinding of outcome assessors was not described  Some of the outcomes were self-reported by the participants |
| Blinding of outcome assessment (detection bias) | Unclear risk | Participants were not blinded  The blinding of outcome assessors was not described  Some of the outcomes were self-reported by the participants  Laboratory testing was performed at the central laboratory |
| Incomplete outcome data (attrition bias) | Low risk | Two participants were withdrawn from the study by investigators  Results of 1 participant were excluded due to “unexplained excessively high concentration of furosemide at 2 min and 5 minutes which were attributed to analytical error” |
| Selective reporting (reporting bias) | Low risk | The full study protocol is unavailable but all pre-specified and expected outcomes of interest are reported |
| Other bias | Low risk | None identified |
| **Gilotra**^15^ **2018** | | |
| Methods | 30 days, randomised, parallel | |
| Participants | 40, all completed the study, mean (SD) age 57 (13) years, NYHA II-IV, median LVEF 25% (IQR 15-55%) | |
| Interventions | Novel preparation of SC furosemide (8 mg/ml) vs. IV conventional furosemide | |
| Outcomes | Urine output at 6 hours: median 1350 ml vs. 1425 ml (p=0.84) | |
| **Risk of bias** |  | |
| **Bias** | **Authors’ judgement** | **Support for judgement** |
| Random sequence generation (selection bias) | Low risk | Participants were randomly allocated in a 1:1 ratio to each treatment arm |
| Allocation concealment (selection bias) | Unclear risk | The randomisation was performed using block-stratified assignments by using a computerised pseudorandom number generator  Personnel performing the randomisation and dispensing the study treatment was blinded to clinical outcomes.  The blinding of outcome assessors not described |
| Blinding of participants and personnel (performance bias) | Unclear risk | Participants were not blinded, personnel performing randomisation was blinded, however blinding of other study personnel was not described |
| Blinding of outcome assessment (detection bias) | Unclear risk | The blinding of outcome assessors was not described  Laboratory testing was performed at the central laboratory  Secondary outcomes included self-reported adverse events e.g. pain, burning, itching |
| Incomplete outcome data (attrition bias) | Low risk | No missing data |
| Selective reporting (reporting bias) | Low risk | The full study protocol is unavailable but all pre-specified and expected outcomes of interest are reported (secondary outcome of hourly urinary output presented as a graph)  Results of follow up not reported |
| Other bias | Low risk | None identified |
| **Sica**^14^ **(FUROPHARM-HF) 2018** | | |
| Methods | 8 hours, randomised, crossover | |
| Participants | 10, all completed the study, mean (SD) age 69.9 (8.6) years, 80% males, all NYHA II | |
| Interventions | Novel preparation of SC furosemide (8 mg/ml) vs. oral conventional furosemide | |
| Outcomes | Urine output at 8 hours: 1833 ml vs. 1550 ml | |
| **Risk of bias** |  | |
| **Bias** | **Authors’ judgement** | **Support for judgement** |
| Random sequence generation (selection bias) | Low risk | Participants were randomly allocated in a 1:1 ratio to each treatment arm  All subjects received the alternate treatment  Participants recruitment and randomisation processes were not described |
| Allocation concealment (selection bias) | Unclear risk | The randomisation process was not described  The blinding of outcome assessors not described |
| Blinding of participants and personnel (performance bias) | Unclear risk | Participants were not blinded  The blinding of outcome assessors was not described |
| Blinding of outcome assessment (detection bias) | Unclear risk | Participants were not blinded  The blinding of outcome assessors was not described  Laboratory testing was performed at central laboratory |
| Incomplete outcome data (attrition bias) | Low risk | All participants completed the study  Results for plasma furosemide levels are presented in a graphic form with a description of the results |
| Selective reporting (reporting bias) | Unclear risk | The full study protocol was unavailable to identify the outcomes  No formal statistical analysis applied  No sample size calculation (“number of participants based on empirical considerations”)  Data presented as mean ± SD (small number of participants, unlikely to be normally distributed) |
| Other bias | Unclear risk | None identified |
| **Sica**^14^ **(PK/PD Pivotal study) 2018** | | |
| Methods | 24 hours, randomised, crossover | |
| Participants | 17, 1 excluded, mean (SD) age 68.0 (9.5) years, 88 males, NYHA II-III | |
| Interventions | Novel preparation of SC furosemide (8 mg/ml) vs. IV conventional furosemide | |
| Outcomes | PK/PD parameters: SC furosemide bioavailability was 99.7% | |
| **Risk of bias** |  | |
| **Bias** | **Authors’ judgement** | **Support for judgement** |
| Random sequence generation (selection bias) | Unclear risk | Participants were randomly allocated in a 1:1 ratio to each treatment arm  One participant did not receive the allocated study treatment and was withdrawn from the study  Participants recruitment and randomisation processes were not described |
| Allocation concealment (selection bias) | Unclear risk | Randomisation process was not described  The blinding of outcome assessors not described  The sequence was predictable for the last participant |
| Blinding of participants and personnel (performance bias) | Unclear risk | Participants were not blinded  The blinding of outcome assessors was not described  Some of the outcomes were self-reported by the participants |
| Blinding of outcome assessment (detection bias) | Unclear risk | Participants were not blinded  Blinding of outcome assessors was not described  Some of the outcomes were self-reported by the participants  Laboratory testing was performed at the central laboratory |
| Incomplete outcome data (attrition bias) | Unclear risk | One participant did not receive allocated treatment and was withdrawn from the study by investigators  Results of 1 participant were excluded due to an “unexpected high concentration of furosemide in a pre-dose sample” potentially affecting the integrity of the trial  Sample size of 16 participants was required, however final analysis included only 15 participants |
| Selective reporting (reporting bias) | Unclear risk | The full study protocol is unavailable but all pre-specified and expected outcomes of interest are reported  Results of follow up not reported  Data presented as mean ± SD (small number of participants, unlikely to be normally distributed) |
| Other bias | Unclear risk | None identified |

IQR- interquartile range; IV- intravenous; NYHA- New York Heart Association; LVEF- left ventricular ejection fraction; PD- pharmacodynamics; PK- pharmacokinetics; SC- subcutaneous; SD- standard deviation
