# Supplementary material for: Subcutaneous furosemide in heart failure: a systematic review
Source: Eur Heart J Cardiovasc Pharmacother. 2024 Nov 8;11(1):94–104. doi: 10.1093/ehjcvp/pvae083 (PMC11805693; doi:10.1093/ehjcvp/pvae083)
Supplement: pvae083_Supplemental_Files [file pvae083_supplemental_files.zip › Supplementary Table 3 clean.docx]

## Table S3. Randomised controlled trials of novel preparations of subcutaneous furosemide: baseline characteristics

| **Trial/ Author/ NCT** | **n** | **Age,**  **mean (years)** | **Men**  **(%)** | **LVEF,**  **median**  **(%)** | **NYHA**  **(%)** | | | **BMI,**  **mean (kg/m^2^)** | **NT-proBNP, median**  **(pg/ml)** | **eGFR,**  **median (ml/min/1.73m^2^)** | **Medications, n (%)** | | | | |
| --- | --- | --- | --- | --- | --- | --- | --- | --- | --- | --- | --- | --- | --- | --- | --- |
|  |  |  |  |  | **II** | **III** | **IV** |  |  |  | **BB** | **ACEi/ARB/ARNi** | **MRA** | **Thiazide diuretic** | **SGLT2i** |
| Konstam  (AT HOME-HF)  2024  US  NCT04593823 | 51 | 64 | 74 | Mean 38 | 6 | 91 | 3 | 36 | Mean 1255 | Mean  55 | 28 (82) | 24 (71) | 8 (24) | 4 (12) | 9 (27) |
| Osmanska^16^  (SQIN-Furosemide PK/PD)  2023  NCT04384653 | 20^^^ | Median  71 | 72 | NR | 89 | 11 | 0 | Median  32 | NR | 68 | 13 (72) | 16 (89) | 4 (22) | NR | 0 |
| Gilotra^15^  2018  NCT02579057 | 40 | 57 | 45 | 25 | 30 | 60 | 10 | 39 | 1551 | Mean  62 | 31 (78) | 23 (58) | 15 (38) | NR | NR |
| Sica^14^  (FUROPHARM-HF) | 10 | 70 | 80 | NR | 100 | 0 | 0 | 28 | 1130 | 54 | NR | NR | NR | NR | NR |
| 2018  NCT02350725 |  |  |  |  |  |  |  |  |  |  |  |  |  |  |  |
| Sica^14^ | 17* | 68 | 88 | NR | 76 | 24 | 0 | 31 | 897 | 63 | NR | NR | NR | NR | NR |
| (PK/PD Pivotal study) |  |  |  |  |  |  |  |  |  |  |  |  |  |  |  |
| 2018  NCT02329834 |  |  |  |  |  |  |  |  |  |  |  |  |  |  |  |

^^^ two participants were withdrawn due to inadequate line priming during subcutaneous infusion

* one participant was withdrawn from the study by investigators before the first administration of the allocated treatment

ACEi- angiotensin-converting enzyme inhibitor; ARB- angiotensin receptor blocker; ARNi- angiotensin receptor-neprilysin inhibitor; BB- beta-blocker; BMI- body mass index; eGFR- estimated glomerular filtration rate; LVEF- left ventricular ejection fraction; MRA- mineralocorticoid receptor antagonist; NR- not reported; NT-proBNP- N-terminal pro-B-type natriuretic peptide; NYHA- New York Heart Association; SGLT2i- sodium-glucose cotransporter-2 inhibitor
