# Supplementary material for: Subcutaneous furosemide in heart failure: a systematic review
Source: Eur Heart J Cardiovasc Pharmacother. 2024 Nov 8;11(1):94–104. doi: 10.1093/ehjcvp/pvae083 (PMC11805693; doi:10.1093/ehjcvp/pvae083)
Supplement: pvae083_Supplemental_Files [file pvae083_supplemental_files.zip › Supplementary Table 4 clean.docx]

Table S4. Non-randomised studies of novel and conventional preparations of furosemide administered subcutaneously: baseline characteristics

| **Trial/ Author** | **n** | **Age, mean (years)** | **Men, n (%)** | **LVEF, mean (%)** | **NYHA, (%)** | | | **BMI, mean (kg/m^2^)** | **NT-pro-BNP, median (pg/ml)** | **eGFR, mean (ml/min/1.73m^2^)** | **Medications, n (%)** | | | | |
| --- | --- | --- | --- | --- | --- | --- | --- | --- | --- | --- | --- | --- | --- | --- | --- |
|  |  |  |  |  | **II** | **III** | **IV** |  |  |  | **BB** | **ACEi/ARB/ARNi** | **MRA** | **Thiazide diuretic** | **SGLT2i** |
| **Non-randomised controlled** | | | | | | | | | | | | | | | |
| Lopez-Vilella^21^  2021 | 27  (10 SC and 17 oral)* | 73 | 8  (80) | 50% had LVEF <40% | 0 | 40 | 60 | NR | Mean  6925 | NR | 5 (50) | 1 (10) | 6 (60) | 1 (10) | 0 |
| Austin^34^  2013 | 25  (11 SC and 14 IV)* | Median 81 | 5  (45) | NR | 0 | 0 | 100 | NR | NR | NR | NR | NR | NR | NR | NR |
| **Prospective observational cohort** | | | | | | | | | | | | | | | |
| Osmanska^16^  SUBCUT-HF I)  2023  NCT04846816 | 20 | Median  75 | 11  (55) | Median 36 | 25 | 70 | 5 | Median  30 | 5184 | 45 | 12 (60) | 13 (65) | 7 (35) | NR | 4 (20) |
| FREEDOM-HF^20^  2023  NCT03458325 | 24 | Median  56 | 15  (63) | NR | NR | NR | 0 | NR | Mean  823 | 61 | 63 | 46 | 42 Spironolactone | 21 | NR |
| Lozano Bahamonde^25^  2018 | 12 | 80 | 9  (75) | 37 | NR | NR | NR | NR | NR | 27 | NR | NR | 5 (42) | 4 (33) | NR |
| **Retrospective observational cohort** | | | | | | | | | | | | | | | |
| Birch^22^  2023 | 116  (130 episodes) | 79 | 86  (66)^£^ | NR | 1 | 31 | 69 | NR | NR | NR | NR | NR | NR | NR | NR |
| Brown^13^  2022 | 28  (36 consecutive episodes) | Median  78 | NR | 57% had LVEF <35% | NR | NR | NR | NR | NR | NR | NR | NR | NR | NR | NR |
| Civera^26^  2022  Spain | 55 | 79 | 32  (58) | Median  48 | 4 | 96 | 0 | NR | 5218 | Median  45 | 51 (93) | 40 (73) | 44 (80) | 46 (87) | 13 (24) |
| Lozano Bahamonde^24^  2019 | 16  (12 with congestive symptoms, 4 euvolaemic*) | 78 | 12  (75) | 37 | 0 | 56 | 44 | NR | NR | NR | NR | NR | 9 (56) | 7 (44) | NR |
| Galindo-Ocana^27^  2013 | 44  (17 SC and 27 IV,  97 episodes)* | Median 84 | 6  (35) | NR | NR | NR | NR | NR | NR | NR | NR | NR | NR | NR | NR |
| Zatarain-Nicolas^23^  2013 | 24  (41 episodes) | 75 | 19  (79) | 58% had LVEF <45% | NR | 93%  NYHA II-IV | NR | 3356 | 17 (73) | 19 (81) | 6 (27) | 5 (20) | NR | NR | NR |
| Zacharias^10^  2011 | 32  (43 consecutive episodes) | NR | 21  (66) | NR | NR | NR | NR | NR | NR | NR | NR | NR | NR | NR | NR |

* characteristics of patients treated with SC furosemide

^£^ reported for all episodes, n=130

ACEi- angiotensin-converting enzyme inhibitor; ARB- angiotensin receptor blocker; BB- beta-blocker; BMI- body mass index; eGFR- estimated glomerular filtration rate; IV- intravenous; LVEF- left ventricular ejection fraction; MRA- mineralocorticoid receptor antagonist; NR- not reported; NT-proBNP- N-terminal pro-B-type natriuretic peptide; NYHA- New York Heart Association; SC- subcutaneous; SGLT2i- sodium-glucose cotransporter-2 inhibitor
