# Supplementary material for: Subcutaneous furosemide in heart failure: a systematic review
Source: Eur Heart J Cardiovasc Pharmacother. 2024 Nov 8;11(1):94–104. doi: 10.1093/ehjcvp/pvae083 (PMC11805693; doi:10.1093/ehjcvp/pvae083)
Supplement: pvae083_Supplemental_Files [file pvae083_supplemental_files.zip › Supplementary Table 5 clean.docx]

## Table S5. Randomised controlled trials of novel preparations of subcutaneous furosemide: inclusion and exclusion criteria

|  | **Trial/ Author/ NCT** | | | | |
| --- | --- | --- | --- | --- | --- |
|  | Konstam^19^  (AT HOME-HF)  2024  NCT04593823 | Osmanska^16^  (SQIN-Furosemide PK/PD)  2023  NCT04384653 | Gilotra^15^  2018  NCT02579057 | Sica^14^  (FUROPHARM-HF)  2018  NCT02350725 | Sica^14^  (PK/PD Pivotal study)  2018  NCT02329834 |
| **Inclusion criteria** | - Age 18 years or older - Diagnosis of symptomatic chronic heart failure (NYHA Class II-IV) with background loop diuretic therapy for at least 4 weeks - Need for augmented diuresis outside of the acute care setting as determined by the investigator - Treated with daily total furosemide equivalent dose (40-160 mg) of loop diuretic or equivalent - Signs of volume expansion, defined as two or more of the following six signs: jugular venous distention, edema (≥ 1+), ascites, pulmonary congestion on chest x-ray, pulmonary rales - NT-proBNP ≥1000 pg/ml (1400 for patients in atrial fibrillation) or, for patients not on Entresto, BNP ≥200 (400 for patients in atrial fibrillation) - Increase over the preceding 30 days in at least one of the following symptoms characteristics of worsening heart failure:   dyspnoea, fatigue, exercise intolerance   - Adequate environment for at home administration of Furoscix by patient or caregiver | - Male and female subjects ≥18 and ≤80 years of age - Weight <130 kg and BMI <38 kg/m^2^ - Female participants: non-pregnant, non-lactating, or post-menopausal, or surgically sterile (e.g., tubal ligation, hysterectomy) or use 2 forms of contraception or abstinence - Chronic HF: treatment for HF ≥3 (NYHA class II/III) with presence of symptoms of chronic volume overload requiring ongoing treatment with oral furosemide at a dose of ≥40 mg per day for at least 30 days prior - Agrees to abstain from using alcohol, caffeine-containing products, and tobacco/nicotine-containing products | - Chronic HF: treatment for HF ≥3 months or recent (60 days) HF hospitalisation - Outpatient with worsening HF requiring IV diuretics - NYHA II-IV - Signs and/or symptoms of fluid overload | - Male and female subjects ≥18 years of age - Weight <120 kg and BMI <30 kg/m^2^ - Female participants: at least 2 years post-menopausal - Treated with oral furosemide (40 mg OD or BD) or therapeutic equivalent (bumetanide 1 mg OD or BD) for a period 90 days - History of chronic HF according to 2012 ESC guidelines | - Male and female subjects ≥18 years of age - Weight <130 kg and BMI <34 kg/m^2^ - Female participants: non-pregnant, non-lactating, and either post-menopausal or surgically sterile or use two forms of contraception or abstinence - Chronic HF: treatment for HF ≥3 (NYHA class II/III) with presence of symptoms of chronic fluid overload requiring ongoing treatment with oral furosemide at a dose of >40 mg per day for at least 30 days prior - Agrees to abstain from using alcohol, caffeine-containing products, and tobacco/nicotine-containing products |
| **Exclusion criteria** | - Suspected high risk clinical instability with outpatient treatment - Presence of a complicating condition, other than heart failure likely to require hospitalization in next 30 days - Pregnant women or women of childbearing age who are not willing to use an adequate form of contraception. - Known allergy to the active and inactive ingredients of the study medication or device adhesive - On experimental medication or currently participating in another interventional research study - eGFR < 20 ml/min/1.73 m^2^ - Serum potassium at baseline > 5.4 mmol/lor < 3.6 mmol/l - Concomitant infection - Heart rate > 110 bpm - Received IV furosemide or bumetanide within last 24 hours | - ADHF or recent history of hospitalisation for HF (prior 4 weeks) - Worsening of signs or symptoms of HF in the 2 weeks prior - Expected to require IV loop diuretics or inpatient treatment for HF - SBP <90 mmHg - Temperature ≥38°C or sepsis or active infection - Serum sodium <130 mmol/l - Serum potassium <3.5 mmol/l - Significant other cardiac abnormalities which may interfere with study participation or study assessments - Current or planned treatment with any IV therapies (including inotropic agents, vasopressors, levosimendan, nesiritide or analogues) or mechanical support (intra-aortic balloon pump, endotracheal intubation, mechanical ventilation, or any ventricular assist device) - Cachexia - Type I diabetes mellitus or Type II diabetes requiring insulin therapy - Presence or need for urinary catheterization, urinary tract abnormality, or disorder interfering with urination - eGFR <45 ml/min/1.73 m^2^ - Moderate-to-severe hepatic dysfunctions - Recent (72 hours) administration of IV radiographic contrast agent or acute contrast-induced nephropathy - Recent (30 days) major surgery - Recent (30 days) administration of an investigational drug or implantation of investigational device, or participation in another trial - Any surgical or medical condition, which in the opinion of the Investigator may pose an undue risk to the subject, interfere with participation in the study, or which may affect the integrity of the study data. - Positive test for hepatitis B surface antigen, hepatitis C, or HIV - Any positive urine drug screen - Concomitant use of any drugs known to interact with furosemide - History of alcohol abuse within 6 months prior - History of severe allergic or hypersensitivity reactions to furosemide. - Recent (30 days) donation of >100 ml of either whole blood or plasma - Recent (4 weeks) possible COVID-19 exposure signs or symptoms of possible COVID-19 infection (including cough, shortness of breath, or temperature ≥ 38°C) - Recent (14 days) travel via airplane or cruise ship | - Signs and symptoms suggestive of high chance of hospitalisation: myocardial ischaemia, uncontrolled arrhythmia, infection, fever >101^o^ F, hemodynamic instability (SBP ≤80 mm Hg, symptomatic hypotension, DBP ≥120 mmHg, or evidence of hypertensive urgency or emergency) - Respiratory compromise - Mental status changes - Acute kidney injury (defined as >25% increase in serum creatinine from baseline) - Hypokalaemia (defined as serum potassium <4.0 mmol/l) - Patients receiving experimental medication therapy or currently participating in another cardiovascular research study - Presence of or need for urinary bladder catheterization - Urinary tract abnormality or disorder interfering with urination - Allergy to loop diuretics | - ADHF or recent history of ADHF or significant worsening in HF symptoms (within prior 2 weeks) - Contraindication to furosemide - SBP <90 mm Hg - Temperature >38°C or sepsis or active infection - Serum sodium <130 mmol/l - Serum potassium <3.0 mmol/l - Current or planned treatment with any IV therapies (including inotropic agents, vasopressors, levosimendan, nesiritide or analogues) or mechanical support (intra-aortic balloon pump, endotracheal intubation, mechanical, ventilation, or any ventricular assist device) - History of gastric or intestinal surgery that may affect absorption of oral medication - Presence or need for urinary catheterization - Current or planned ultrafiltration, hemofiltration, or dialysis - eGFR <30 ml/min/1.73 m^2^ - Recent (72 hours) administration of IV radiographic contrast agent or acute contrast-induced nephropathy - Recent (30 days) major surgery - Recent (30 days) administration of an investigational drug or implantation of investigational device, or participation in another trial - Inability to follow instructions or comply with procedures - Any surgical or medical condition which in the opinion of the investigator may interfere with participation in the study or which may affect the outcome of the study | - ADHF or recent hospitalisation for HF (prior 4 weeks) - Worsening of signs or symptoms of HF (prior 2 weeks) - Expected to require IV loop diuretics or in-patient treatment for HF - History of chronic skin conditions requiring medical therapy - SBP <90 mmHg - Temperature >38°C or sepsis or active infection - Serum sodium <130mmol/l - Serum potassium <3.0 mmol/l - Significant other cardiac abnormalities that may interfere with study participation or study assessments - Current or planned treatment with any IV therapies (including inotropic agents, vasopressors, levosimendan, nesiritide or analogues) or mechanical support (intra-aortic balloon pump, endotracheal intubation, mechanical ventilation, or any ventricular assist device) - Type I diabetes mellitus or Type II diabetes requiring insulin therapy - Presence or need for urinary catheterization, urinary tract abnormality, or disorder interfering with urination - eGFR <45 ml/min/1.73m^2^, - Moderate-to-severe hepatic dysfunctions - Recent (72 hours) administration of IV radiographic contrast agent or acute contrast-induced nephropathy - Recent (30 days) major surgery - Recent (30 days) administration of an investigational drug or implantation of investigational device, or participation in another trial - Any surgical or medical condition which in the opinion of the investigator may interfere with participation in the study or which may affect the outcome of the study - Positive test for hepatitis B, hepatitis C, or HIV - Positive urine drug screen - Concomitant use of any drugs known to interact with furosemide - History of alcohol abuse within 6 months prior - History of severe allergic or hypersensitivity reactions to furosemide - Recent (30 days) donation of >100 ml of either whole blood or plasma |

BD- twice daily; BMI- body mass index; DBP- diastolic blood pressure; eGFR- estimated glomerular filtration rate; ESC- European Society of Cardiology; HF- heart failure; HIV- human immunodeficiency virus; IV- intravenous; NYHA- New York Heart Association; OD- once daily; SBP- systolic blood pressure
