# Supplementary material for: Subcutaneous furosemide in heart failure: a systematic review
Source: Eur Heart J Cardiovasc Pharmacother. 2024 Nov 8;11(1):94–104. doi: 10.1093/ehjcvp/pvae083 (PMC11805693; doi:10.1093/ehjcvp/pvae083)
Supplement: pvae083_Supplemental_Files [file pvae083_supplemental_files.zip › Supplementary Table 6 clean.docx]

Table S6. Adverse events reported in the studies of furosemide administered subcutaneously in patients with heart failure.

| **First author/**  **Trial/**  **Year/Country/NCT** | **n** | **Adverse events and skin reactions related to treatment with SC furosemide** |
| --- | --- | --- |
| Novel preparations of furosemide | | |
| Konstam^19^  (AT HOME-HF)  2024  US  NCT04593823 | 51 | 21% experienced hypokalaemia  15% experienced renal impairment  15% experienced infusion site pain  3% experienced fatigue  1 patient admitted to hospital with dehydration  1 patient developed hypomagnesaemia |
| FREEDOM-HF^20^  2023  US  NCT03458325 | 24 | 2 episodes of haemorrhage  7 episodes of bruising  7 episodes of infusion site pain |
| Osmanska^16^  (SQIN-Furosemide PK/PD)  2023  NCT04384653 | 20 | 5 episodes of pain or discomfort during treatment  4 episodes of localised skin reaction |
| Osmanska^16^  SUBCUT-HF I)  2023  NCT04846816 | 20 | 4 episodes of localised skin reaction |
| Gilotra^15^  2018  US  NCT02579057 | 40 |  |
| Sica^14^  (FUROPHARM-HF)  2018  US  NCT02350725 | 10 | Following events reported for both studies together:  9 episodes of erythema  6 episodes of swelling |
| Sica^14^  (PK/PD Pivotal study)  2018  US  NCT02329834 | 17 |  |
| Conventional preparations of furosemide | | |
| Birch^22^  2023  UK | 116 (multiple episodes) | 11 episodes of erythema  7 mild adverse events, details not described  5 dislodged access  4 site infections  1 bleeding  1 swelling  1 trauma  1 leaking access |
| Brown^13^  2022  UK | 28 (multiple episodes) | 2 hospitalisations due to inability to tolerate subcutaneous infusion  1 skin infection requiring treatment with antibiotics |
| Civera^26^  2022  Spain | 55 | 1 skin infection requiring treatment with antibiotics |
| Lopez-Vilella^21^  2021  Spain | 27 | 1 localised skin irritation |
| Lozano Bahamonde^24^  2019  Spain | 16 | 2 skin erosions without infection  2 skin infections requiring treatment with antibiotics |
| Lozano Bahamonde^25^  2018  Spain | 12 | 2 skin infections requiring treatment with antibiotics  1 treatment withdrawal due to complications (not specified) |
| Austin^34^  2013  Australia | 25 |  |
| Galindo-Ocana^27^  2013  Spain | 44  (multiple episodes) | 3 haematomas  1 skin infection requiring treatment with antibiotics |
| Zatarain-Nicolas^23^  2013  Spain | 24 (multiple episodes) | 7 skin reactions/abscess  12 skin irritations  10 disconnections/line kinking |
| Zacharias^10^  2011  UK | 32  (multiple episodes) | 8 mild skin reactions  1 skin reaction requiring treatment with antibiotics  1 recurrent site reaction |
